# CTCF-Mediated and *Pax6*-Associated Gene Expression in Corneal Epithelial Cell-Specific Differentiation

**DOI:** 10.1371/journal.pone.0162071

**Published:** 2016-09-01

**Authors:** Shanli Tsui, Jie Wang, Ling Wang, Wei Dai, Luo Lu

**Affiliations:** 1 Department of Medicine, David Geffen School of Medicine, University of California Los Angeles, Torrance, CA, 90502, United States of America; 2 Department of Environmental Medicine, New York University School of Medicine, Tuxedo, NY, 10987, United States of America; Università degli Studi di Milano, ITALY

## Abstract

**Background:**

The purpose of the study is to elicit the epigenetic mechanism involving CCCTC binding factor (CTCF)-mediated chromatin remodeling that regulates *PAX6* gene interaction with differentiation-associated genes to control corneal epithelial differentiation.

**Methods:**

Cell cycle progression and specific keratin expressions were measured to monitor changes of differentiation-induced primary human limbal stem/progenitor (HLS/P), human corneal epithelial (HCE) and human telomerase-immortalized corneal epithelial (HTCE) cells. *PAX6*-interactive and differentiation-associated genes in chromatin remodeling mediated by the epigenetic factor CTCF were detected by circular chromosome conformation capture (4C) and ChIP (Chromatin immunoprecipitation)-on-chip approaches, and verified by FISH (Fluorescent in situ hybridization). Furthermore, CTCF activities were altered by CTCF-shRNA to study the effect of CTCF on mediating interaction of Pax6 and differentiation-associated genes in corneal epithelial cell fate.

**Results:**

Our results demonstrated that differentiation-induced human corneal epithelial cells expressed typical corneal epithelial characteristics including morphological changes, increased keratin12 expression and G_0_/G_1_ accumulations. Expressions of CTCF and PAX6 were suppressed and elevated following the process of differentiation, respectively. During corneal epithelial cell differentiation, differentiation-induced *RCN1* and *ADAM17* were found interacting with *PAX6* in the process of CTCF-mediated chromatin remodeling detected by 4C and verified by ChIP-on-chip and FISH. Diminished CTCF mRNA with CTCF-shRNA in HTCE cells weakened the interaction of *PAX6* gene in controlling *RCN1/ADAM17* and enhanced early onset of the genes in cell differentiation.

**Conclusion:**

Our results explain how epigenetic factor CTCF-mediated chromatin remodeling regulates interactions between eye-specific *PAX6* and those genes that are induced/associated with cell differentiation to modulate corneal epithelial cell-specific differentiation.

## Introduction

Corneal epithelial layer integrities are maintained by continuous processes of self-renewal and wound healing. Both the self-renewal and wound-healing processes are affected by stimulation of growth factors and environmental stresses that activate cellular signaling pathways and transcription factors to switch the stimulatory signals to genetic responses [1,2,3,4,5,6,7,8]. For an example, the effect of EGF on suppressing eye-specific Pax6 transcription in proliferation of corneal epithelial cells is regulated through activation of an epigenetic regulator termed CCCTC binding factor (CTCF) [9] [10]. CTCF is a highly conserved zinc finger (ZF) protein in mammalian cells to epigenetically control cellular physiological processes [9]. CTCF regulates DNA imprinting, X chromosome inactivation and transcriptional control of various gene expressions, including *p19ARF*, *p16INK4a*, *PIM-1*, *PLK*, *BRCA1*, *p53*, *p27*, *Ecadherine*, *E2F1*, *TERT* and *PAX6* [10,11,12,13,14]. Activities of CTCF insulator-function are often located between the boundaries of gene enhancers and promoters to regulate the chromatin’s effect on flanking regions, which is often modified by a DNA methylation (CH_3_)-sensitive process [15,16,17]. Genome-wide analyses have revealed that CTCF is able to bind tens of thousands of DNA sites using different combinations of its eleven zinc fingers involving intra- and inter-chromatin interactions. Emerged evidence indicates that CTCF plays a master role in genomic spatial organization and mediates these extensive long- range intra- and inter-chromatin interactions [9]. One of the most significant epigenetic roles in chromatin remodeling of CTCF is to mediate environmental signals and cooperatively introduce cell-type specific inter-chromatin interactions leading to specific gene expression [18,19,20,21]. In corneal epithelial and retinoblastoma cells, CTCF controls *PXA6* transcription by interacting with a repressor element located in the 5’-flanking region upstream from the *PAX6* P0 promoter. This interaction suppresses *PAX6* transcription by blocking an ectoderm enhancer (EE) located approximately -3.5 kb upstream from the P0 promoter [13]. However, important epigenetic question, concerning whether CTCF-mediated chromatin remodeling affecting interactions of *PAX6* with other cell differentiation-related genes in eye-specific expression, is still unresolved.

Homeobox transcription factor Pax6 is an important member in the *PAX* family and plays a critical role in eye and neuronal development in both vertebrates and invertebrates [22]. PAX6 is expressed essentially in all ocular structures, including the cornea, iris, lens and retina [23,24,25,26]. PAX6 plays important roles in promoting corneal epithelial and neuron apoptosis [27,28]. Regulation of *PAX6* gene transcription is highly conserved during evolution. In most species, *PAX6* transcription is regulated via two promoters, P0 and P1 [29,30,31]. There is a highly conserved transcriptional control element termed ectoderm enhancer (EE) that is located approximately -3.5 kbp upstream from the P0 promoter [32]. It has shown that a repressor element composed of 80-bp nuclear acids that is located about 1.2 kb upstream from the P0 promoter of *PAX6* gene. It contains five functional CCCTC motifs in this region [14]. CTCF regulates PAX6 activities in response to growth factor and stress stimulation. For instance, epidermal growth factor (EGF)-induced suppression of Pax6 expression by CTCF is required in corneal epithelial proliferation [33]. In contrast, ultraviolet (UV) stress inhibits CTCF expression and minimizes CTCF DNA binding activity to the repressor element in the *PAX6* gene [14]. In transgenic mice over-expressing CTCF, *PAX6* is decreased its expression resulting in retardation of embryonic ocular development including the cornea, lens and retina [13,34]. In addition, DNA methylation plays a role in CTCF-controlled PAX6 expression during mouse ES cell differentiation to further provide the regulatory mechanism of PAX6 in early stage ES and progenitor cells. In the present study, the effect of CTCF-mediated chromatin remodeling on *PAX6* and differentiation-associated genes was investigated to demonstrate how eye-specific *PAX6* interacts in the promoter regions with differentiation-associated genes, such as *RCN1* and *ADAM17* during corneal epithelial cell differentiation. Taken together, as one of the important chromatin architecture organizers, CTCF participates in gene transcriptional regulation in corneal epithelial cells. Our data reveal a novel mechanism involving CTCF-mediated chromatin remodeling that regulates interactions between eye-specific *PAX6* gene and differentiation-associated genes to modulate corneal epithelial differentiation.

## Methods

### Corneal epithelial cell culture

*Human telomerase-immortalized corneal epithelial (HTCE) cells*. HTCE cell line was originated by laboratory of J.V. Jester [35]. HTCE cells were cultured in defined keratinocyte-SFM medium containing 90 μM calcium in a humidified incubator at 37°C with 5% CO_2_. Differentiation of HTCE cells was induced by adding 1.2 mM calcium and 5% FBS in the culture medium. *Human limbal stem/progenitor (HLS/P) cell culture*. Human sclerocorneal tissues were obtained from Illinois Eye Bank (Watson Gailey, Bloomington, IL) and Lions Eye Institute for Transplant and Research (Tampa, FL). Human tissue was handled in accordance with the tenets of the Declaration of Helsinki. The experimental protocol was exempted by the University of California Los Angeles Institutional Review Board. The sclerocorneal rim tissue was incubated in 2.4 U/mL of dispase II (Roche, Indianapolis, IN) at 37°C for 2 h in DMEM/F-12 (Ham) medium (Life Technologies, Carlsbad, CA) with 5% of fetal bovine serum (FBS; Life Technologies). The epithelial cell sheet was isolated by gentle scrapping under the dissecting microscope and incubated with 0.25% trypsin-1 mM EDTA (Life Technologies) for 5 min to achieve a single-cell suspension. HLS/P cells were cultured at the density of 300 cells/cm^2^ on a monolayer of mitomycin C (Sigma-Aldrich, St. Louis, MO) growth-arrested 3T3-J2 mouse fibroblasts (Howard Green Lab, Harvard Medical School) at the density of 3 x 10^4^ cm^2^ in supplemental hormone epithelial medium (SHEM) consisting of DMEM/F-12 (Ham) medium supplemented with 5% FBS, N2 supplement (Life Technologies), 2 ng/mL of epidermal growth factor (Life Technologies), 8.4 ng/mL of cholera toxin (Sigma-Aldrich), 0.5 g/mL of hydrocortisone (Sigma-Aldrich) and 0.5% of dimethyl sulfoxide (Sigma-Aldrich) at 37°C under 5% CO_2_ for 14 days. *Human corneal epithelial (HCE) cells*. Primary HCE cells were obtained by passing HLS/P cells into collagen/ fibronectin (50/50%) coated surface and an airlift (reduced medium level) procedure to induce differentiation. HCE cells were grown in a serum-free Defined Keratinocyte medium (Invitrogen, CA) in an incubator supplied with 95% air and 5% CO_2_ at 37°C. The morphological changes were closely monitored.

### Lv-control and CTCF-shRNA cells, and cell cycle analysis

CTCF activity in HTCE cell was altered by knocking down its expression with lentiviral particles, containing shRNA of CTCF tagged with a variant of green fluorescent protein (Turbo-GFP; Sigma-Aldrich, St. Louis, MO) as described in previous publication [36]. Briefly, lentiviral particles, were packaged in HEK-293T cells by co-transfection of HEK-293T cells with pCMV-VSV-G, psPAX2, and pGIPZshRNA-CTCF fused to the GFP for 72 h (Open Biosystems, Huntsville, AL). The culture media containing lentiviral particles secreted from HEK-293T cells were freshly added to HTCE cells, and infected cells stably expressing shRNAs/GFP were selected as a single clone in selective culture medium with puromycin (2 μg/ml). HTCE cells infected with a lentiviral particle containing pGIPZ-control vector served as the control cells (termed as Lv-control).

Cell cycle analysis was performed using a flow cytometry (BD LSRII, BD Biosciences, San Jose, CA). HTCE cells were treated with calcium (1.2 mM) and FBS (5%) following a time course as indicated, and then the attached cells were trypsinized and fixed with 70% ethanol and 50 mM glycine. The cells were re-suspended in PBS containing RNase A (100 ng/ml) and propidium iodide (PI, 25 ng/ml). Cell populations in different phases were mapped with a BD FAC Diva Software V6.11, and cell cycle progression were analyzed with MedFit LT^TM^ V3.1 (Verity Software House). Statistical analysis was performed as described in the statistical section below.

### Circularized Chromosome Conformation Capture (4C) and microarray

Methods for 4C experiments were developed and modified according to a publication by Simonis et al. [37]. Briefly, DNA-protein complexes were cross-linked in 1% formaldehyde at room temperature (RT) for 10 minutes, purified and digested with HindIII to separate the non-cross-linked DNAs from the cross-linked ones. The reason to choose HindIII is because there is HindIII cutting site on either side of CTCF binding sites 2 and 3 in the region of *PAX6* promoter. These digested fragments were ligated intramolecularly in the presence of T4 DNA ligase at 16°C for overnight. The process to reverse cross-link was performed in a high temperature treatment for 10 min. These ligated DNA fragments were further cleaved with a frequent cutter DpnI, and were subsequently re-ligated to form small DNA circles. The pool of these circular fragments became the 4C product library and small captured fragments in the library were amplified by inverse PCR using bait-specific primers in experiments facing outward. These paired primers were designed to cover two (sites 2&3) of the five predicted CTCF zinc finger protein-binding sites in promoter region of Pax6 [14,38]. These sites were composed of multiple CCCTC nucleotide repeats in the promoter region according to human Pax6 DNA sequence. These PCR products were used for characterization of target genes on a NimbleGen human ChIP-chip 2.1M deluxe promoter array manufactured by Roche. The bioinformatics analysis for 4C and DNA microarray data was conducted by using DAVID Bioinformatics resources v6.7 (The Database for Annotation, Visualization and Integrated Discovery through the web site: http://david.abcc.ncifcrf.gov).

### Fluorescence in situ hybridization (FISH)

FISH assays were performed to determine position of *PAX6* gene locus relative to both *RCN1* and *ADAM17* gene loci. The bacterial artificial chromosome (BAC) clones RPCI-11-26B16 encompassing *PAX6* gene was labeled with Red 5-ROX dUTP (Empire genomics). Other BACs RPCI-11-122P23 for *RCN1* and RPCI-11-257F10 for *ADAM17* were labeled with Green 5-Fluorescein dUTP (Empire genomics). These labeled DNAs were served as hybridization probes in FISH assays. HTCE cells grown on multi-chamber slides were fixed with MAA fixative (methanol/ acetic acid, 3:1) for 10 min at room temperature. The slides were dehydrated and denatured in denaturation buffer (70% formamide, 2XSSC, pH7.0–8.0) at 73°C for 5 min, and the probes were also denatured at the same temperature for 5 min. Hybridizations were performed in dark at 37°C overnight. Slides were washed with 2XSSC/NP40 and counterstained with Dapi containing anti-fade reagents. Images were observed and photographed by Zeiss AXIO microscope. At least 100 cells were observed and analyzed in each treatment of FISH assay.

### Chromatin immunoprecipitation assay (ChIP)

HTCE cells were treated as indicated and cross-linked by incubating them with 1% formaldehyde for 10 min at room temperature (RT). Furthermore, cells were lysed before sonication to shear chromatin to an average length of 200 bp to 1 kb. Resulted cell lysates were immuno-precipitated with antibody specific to CTCF (Cell signaling). Salmon sperm DNA treated Protein A agarose beads (Millipore) were included to collect the antigen-antibody complex which was washed later to remove any non-specific binding. DNA was eluted from the beads using 0.1 M NaHCO_3_ and 1% SDS and ready for PCR reactions by using specific primer sets spanning CTCF binding sites of various promoters. Those specific primer sets include *PAX6* site 2 with forward 5’-ccccggctagcccccaacc-3’ and reverse 5’-agaggaacccgcggaggagaggat-3’; *PAX6* site 3 with forward 5’-cgcggtgacataattacctctgac-3’ and reverse 5’-gcgggaaagtttgtgcagcgagag-3’; *RCN1* site 2 with forward 5’-tcactgagaaaacgcacacc-3’ and reverse 5’-acgagcactgcatccaaaac-3’; and *ADAM17* site 2 with forward 5’-gagccggcctttggtaac-3’ and reverse 5’-cctagcccctcaatcctctt-3’.

### Immunostaining of corneal epithelial cells

For immunostaining experiments, corneal epithelial cells were fixed for 15 min in 4% paraformaldehyde, and then permeabilized with PBS-0.2% triton-X100 (PBS-T) for 30 min at RT. The cells were blocked by incubation with 10% normal horse serum (NHS) or 10% normal goat serum in PBS-T for 1 h at RT, followed by immunostaining with the corresponding antibodies. Corneal epithelial cells on slices were washed with PBS and stained with DAPI. A Nikon fluorescent Ti microscope was used to capture stained tissue imaging. Imaging data were analyzed using a Nikon NIS Element Software program.

### Western blot and quantitative RT-PCR

Cells were treated following a time course as indicated and harvested in appropriate amount of Lamili SDS sample buffer with DTT for Western blot. Cell lysates were boiled and cleared by centrifugation at 13,000xg for 2 min. Equal amounts of the protein samples were fractionated by electrophoresis with 7% and 15% SDS-PAGE gels. The fractionated proteins were transferred onto PVDF membranes, probed with primary antibodies against K12 (as a gift from Dr. Winston Kao’s lab at University of Cincinnati), CTCF (Cell signaling) and Pax6 (Santa Cruz Biotechnology, Santa Cruz, CA). HRP-conjugated secondary antibodies were applied at 1:1000 dilution and targeted protein bands were visualized using Western blotting Luminol Reagent (Santa Cruz Biotechnology, CA). The same cell treatments were performed as indicated and lysed in appropriate amount of buffer RP1 with beta-mercaptoethanol. Total RNAs were extracted from these lysates according to the manufactural protocol (Clontech). Integrity of RNAs was analyzed using Bioanalyzer (Agilent Technologies, Inc). Reverse transcription and PCR were performed as described previously. Primer sets used for RT-PCR are listed as follows: for K12 forward 5’-caatgcgagactagctgctg-3’ and reverse 5’-gctcatcctcgtggttcttc-3’; for *PAX6* forward 5’-gagtgcccgtccatctttg-3’ and reverse 5’-gtctgcgcccatctgttgcttttc-3’; for *CTCF* forward 5’-ccctgcggcttttgtctgttctaa-3’ and reverse 5’-ctgtttgggctggttggttctgc-3’; for *RCN1* forward 5’-ctaggaaaccccgcagagtttca-3’ and reverse 5’-gatgtcctccagggtttccaa-3’; and *ADAM17* forward 5’-tgcagtgacaggaacagtcc-3’ and reverse 5’-ggatgcatttcccatcctta-3’.

### Statistical analysis

For Western analysis, signals in the films were scanned digitally and optical densities (OD) were quantified by using the Image Calculator software. The relative OD was calculated by normalizing the signals from target proteins against intensities of loading controls. All of the experimental data were subjects to statistical analysis and plotted as Mean±SE. Significant differences between the control and treated groups were determined by One-way ANOVA, Dunnett and Tukey’s tests (*F<0*.*05)*, respectively. Student’s *t* test was used to determine the significant difference for paired samples at *P*<0.05.

### Data access

The raw data files generated by promoter microarray analysis have been submitted to Gene Expression Omnibus (GEO, http://www.ncbi.nlm.nih.gov/geo/query/acc.cgi?acc=GSE84295). Reference Series Number for the data set is GSE84295.

## Results

### Increases in expression of keratins during corneal epithelial cell differentiation

**Fig 1:**
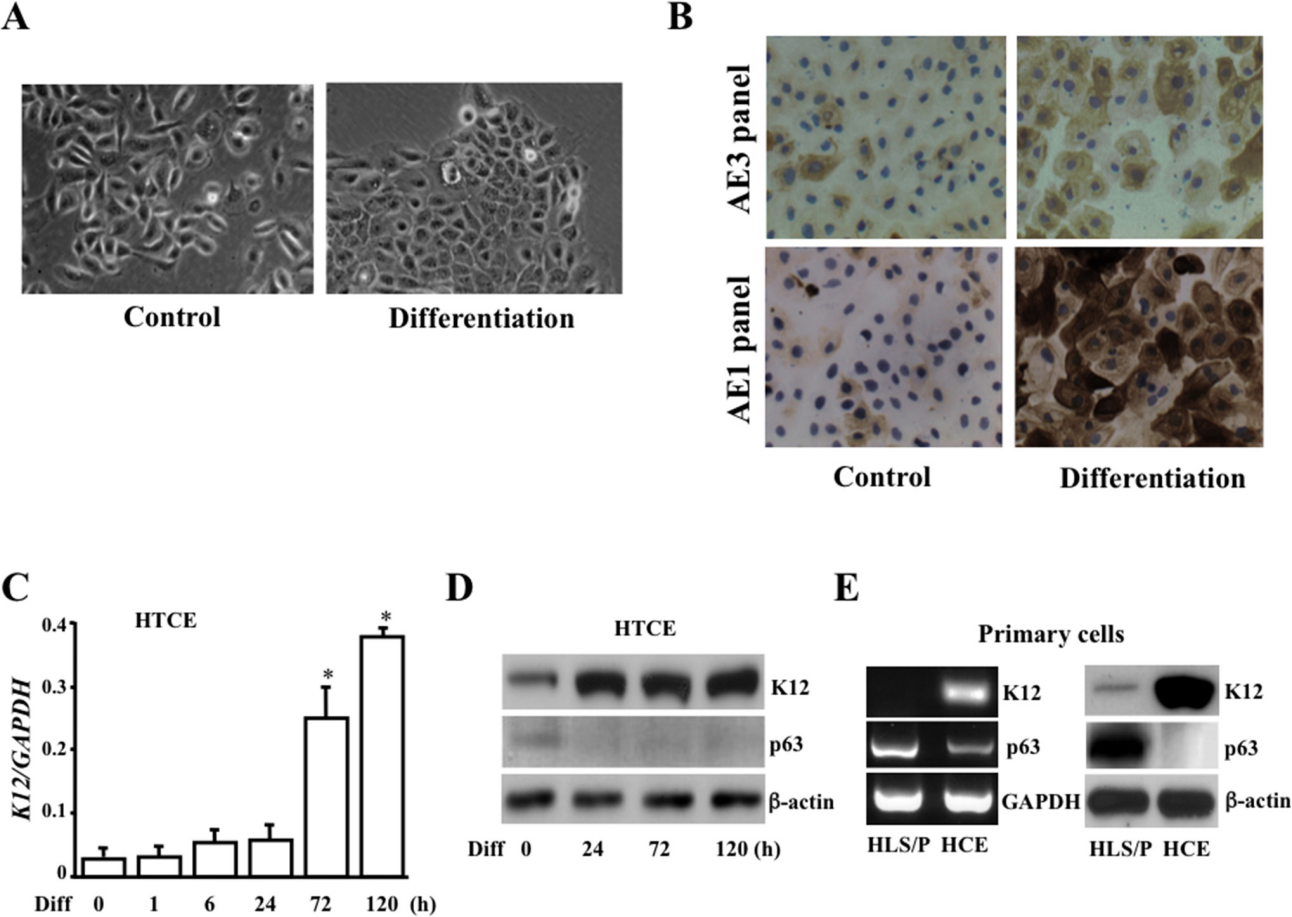
Characterization of differentiation-induced corneal epithelial cells. (***A***) Morphological changes in culture during differentiation of HTCE cells. (***B***) AE3 and AE1 keratin panel staining in HTCE cell differentiation. (***C***) Time course of K12 mRNA expression following induced differentiation of HTCE cells. Differentiation of HTCE cells was induced by adding 1.2 mM calcium and 5% FBS in the normal culture condition up to 120 h. (***D***) Detection of K12 and p63 expression in HTCE cell differentiation by Western blot up to 120 h. (***E***) Expression of K12 and p63 mRNAs and proteins in human limbal stem/progenitor (HLS/P) and corneal epithelial (HCE) cells. Cells were grown and induced to differentiation in chamber slides and photos were taken by light microscopy. These cells were then washed in PBS and fixed with 95% ethanol before staining. Staining protocols were used as suggested by manufactures. Furthermore, RNAs were extracted from HTCE, HLS/P and HCE cells, and reverse-transcribed into cDNAs before real-time qPCR analysis was performed using K12-specific primers as indicated in Materials and Methods. Symbol “*” indicates significant differences after 72 h induction (*p*<0.05, n = 6). Photos were taken by a Zeiss AXIO microscope (x20).

To study CTCF-mediated *PAX6* and gene regulations, HTCE cells were induced toward terminal differentiation by increasing calcium concentration and adding FBS in the normal culture conditions. In differentiated HTCE cells, cell morphology was changed by expressing more characteristics for monolayer epithelial cells, which includes changing cellular nucleus/plasma ratio, becoming flat and forming clear tight adhesions (*Fig 1A*). Expression of various epithelial cell-specific keratins was markedly increased in differentiation-induced HTCE cells by staining the cells with both AE3 and AE1 epithelial cell differentiation panels, suggesting HTCE cells indeed committed to differentiation after induction (*Fig 1B*). More importantly, expression of human corneal epithelial cell-specific keratin 12 (K12) was significantly increased in mRNA levels in differentiation-induced HTCE cells following a time course (*Fig 1C*). Protein expressions of K12 and p63 were examined by Western analysis in differentiation-induced HTCE cells (*Fig 1D*). To further verify altered expression of K12 and p63 in progenitor and differentiation-induced corneal epithelial cells, mRNA and protein levels were detected in primary cultured HLS/P and HCE cells by RT-PCR and Western analysis (*Fig 1E*). These results demonstrate that human progenitor and HTCE cells induced by calcium and FBS were differentiated toward corneal epithelial cells evidenced by decrease in p63 expression and increases in expressions of multiple epithelial keratins including human corneal epithelium-specific K12.

### Altered cell cycle distribution and CTCF/PAX6 expression during cell differentiation

**Fig 2:**
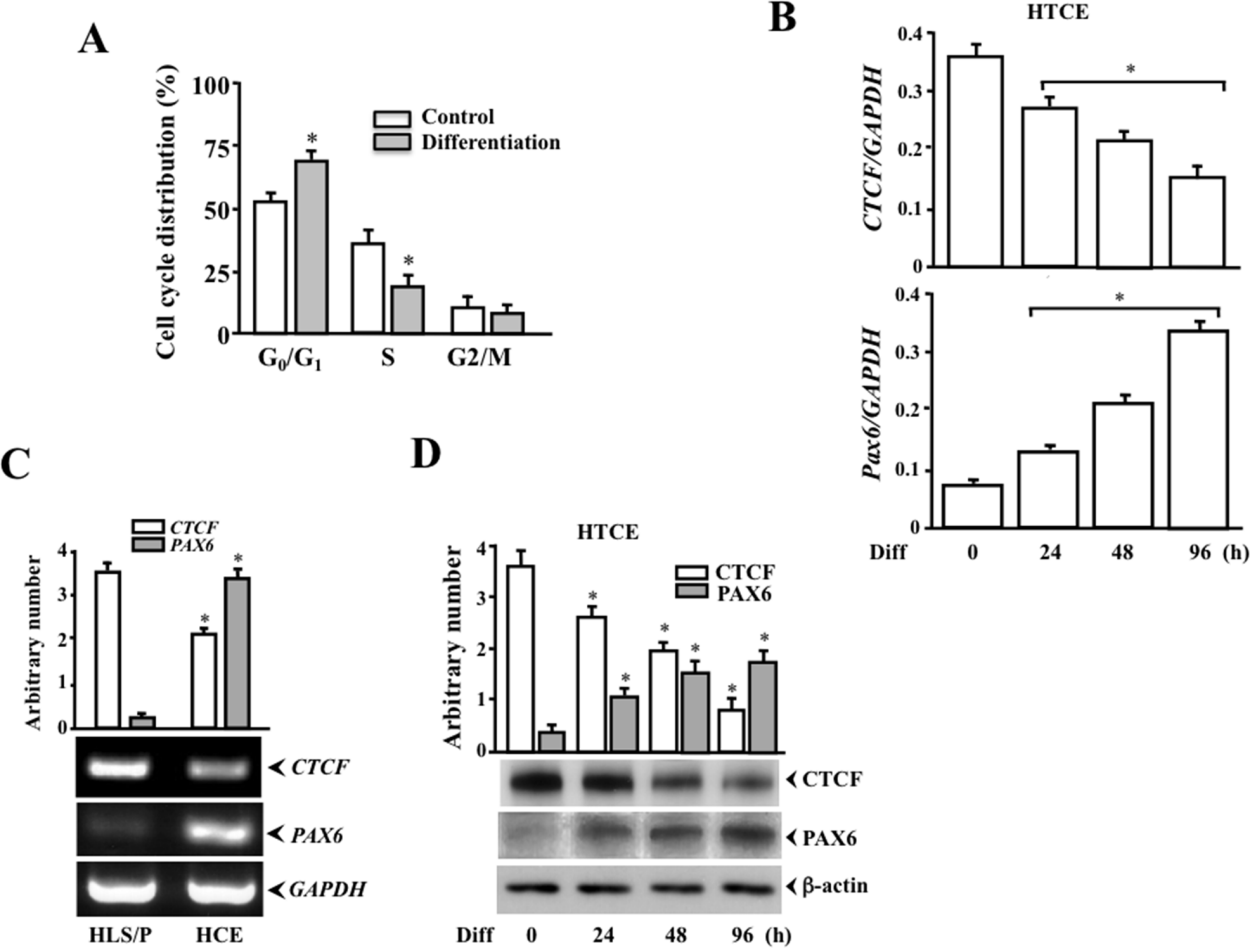
Correlation of CTCF and PAX6 expression during corneal epithelial cell differentiation. (***A***) Cell cycle analysis by flow cytometry revealed significantly increased and decreased cell populations in G_0_/G_1_ and S phases, respectively. (***B***) Time courses of CTCF and *PAX6* mRNA expressions in HTCE cell differentiation. (***C***) Detection and analysis of CTCF and *PAX6* mRNA expressions in HLS/P and HCE cells. (***D***) Western analysis demonstrated that there is an opposite expression pattern between CTCF and Pax6 following a time course of HTCE cell differentiation (Diff). Flow cytometric analysis of HTCE cells with or without differentiation was performed as described in materials and methods section. Expressions of CTCF and Pax6 in both their protein and RNA levels were detected by Western blots and quantitative real-time RT-PCR during HTCE cell differentiation for as long as 96 h after induction. Symbol “*” indicates significant differences (*p*<0.05, n = 4).

Recent studies demonstrate that over-expression of PAX6 in stem/progenitor cells induces a high expression of nestin, suggesting that PAX6 is involved in stem cell differentiations toward neural progenitor cells [39,40,41]. To study the effect of CTCF-regulated PAX6 activity in HTCE cell differentiation, cell cycle distribution was compared before and after differentiation among wild type and later CTCF-shRNA HTCE cells. First, there were significant increased and decreased cell populations in G_0_/G_1_ phase and S phase during differentiation, respectively (*Fig 2A*). Expression of mRNA levels for *CTCF* and *PAX6* were detected by RT-PCR following a 96 h time course of HTCE cell differentiation (*Fig 2B*). RT-PCR was also performed to detect CTCF and *PAX6* mRNA expression in HLS/P and HCE cells and to verify the effect of differentiation on changes of CTCF and *PAX6* levels. Expressions of CTCF and PAX6 protein levels analyzed by Western blots were gradually declined and increased following a time course of HTCE cell differentiation up to 96 h, respectively (*Fig 2D*). Expression patterns of *CTCF* and *PAX6* mRNA levels in differentiation-induced HTCE cells were consistent with their protein expression levels. These observations were consistent to the results found in our previous studies that show Pax6 plays functional roles in differentiation of both primary and SV-40 transformed human corneal epithelial cells.

### Effect of CTCF-mediated chromatin interactions on *PAX6* and its associated genes

**Fig 3:**
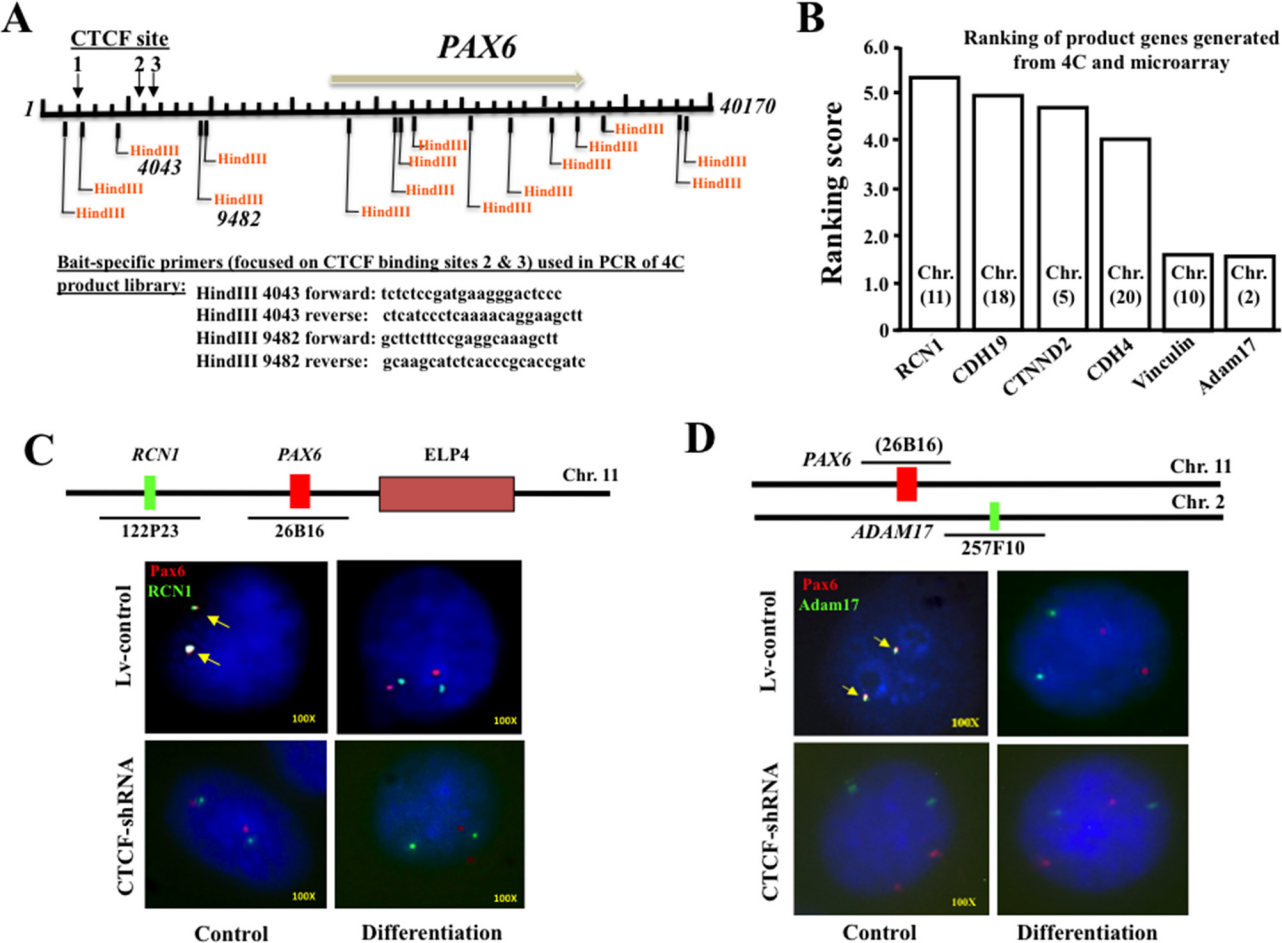
CTCF-mediated chromatin interactions of *PAX6* with *RCN1* and *ADAM17* genes. (***A***) Illustration of circularized chromosome conformation capture (4C) primers designs relative to HindIII restriction sites which are adjacent to CTCF binding sites 2 and 3 on *PAX6* promoter. (***B***) Six product genes were listed as CTCF-mediated *PAX6* interactive genes (score >1.5) from analysis with 4C and microarray results. (***C***) Confirmation of 4C results by FISH to truly identify interaction in promoter regions of *PAX6* and *RCN1* genes in lentiviral infected control (Lv-control), and lentiviral CTCF-shRNA infected (CTCF-shRNA) and CTCF-knocked down HTCE cells. (***D***) Verification of 4C technology by FISH to truly identify interaction in promoter regions of *PAX6* and *ADAM17* genes in Lv-control and CTCF-shRNA cells. Details of 4C and use of microarray were discussed in materials and methods. FISH assays were performed as described in materials and methods to determine positions of *PAX6* gene locus relative to both *RCN1* and *ADAM17* gene loci. The BAC clones RPCI-11-26B16 encompassing *PAX6* gene was labeled with Red 5-ROX dUTP (Empire genomics). Other BACs RPCI-11-122P23 for *RCN1* and RPCI-11-257F10 for *ADAM17* were labeled with Green 5-Fluorescein dUTP (Empire genomics).

The effect of chromatin interactions on *PAX6* and *PAX6*-associated genes were detected by circularized chromosome conformation capture (4C) with CTCF binding site-specific primers. In 4C experiments (as 4C protocols were described in details in methods section), two pairs of primers used to amplify product library were designed according to locations of the HindIII restriction sites on either side of predicted CTCF binding sties 2 and 3 in the promoter region of human Pax6 gene (*Fig 3A*). Potential Pax6-associated PCR products from this CTCF-specific 4C experiments were further used in DNA genome microarray to identify *PAX6*-associated genes in HTCE cell differentiation. Some examples of the specific genes interacting with *PAX6* in the promoter regions with relatively higher scores (>1.5 times) were listed in *Fig 3B*. In this study, we focused on both *RCN1* (scored 5.4) from chromosome 11 and *ADAM17* (scored 1.6) from chromosome 2, which are among the Pax6-associated genes involving cell differentiation. Furthermore, we verified the results obtained in 4C technology with FISH experiments. By combining with microscopy technique, FISH has an advantage to provide visual resolution for observations of chromatin remodeling processes. In addition, CTCF-knockdown HTCE cells were also included in FISH. It has been shown that results from FISH experiments can confirm the results from 4C technology and genome microarray. The CTCF-mediated chromatin interaction for *PAX6* and *RCN1* (indicated by arrows) from the same chromosome 11 was altered by cell differentiation in CTCF activity altered cells termed as Lv-control and CTCF-shRNA cells. Changes of CTCF-mediated chromatin interaction for *PAX6* and *RCN1* were only LV-control cells, but not observed in CTCF-knocked down CTCF-shRNA cells (*Fig 3C*). FISH experiments also confirmed the CTCF-mediated chromatin interaction for Pax6 and Adam17 from chromosome 11 and 2, respectively (indicated by arrows) and consequently disassociated during differentiation (*Fig 3D*). However, there were no changes found in lenti-viral CTCF-shRNA infected cells. These results indicate that CTCF-mediated chromatin remodeling of *PAX6* and *PAX6*-associated genes, such as *RCN1* and *ADAM17*, are indeed occurred in the process of HTCE cell differentiation.

### Effect of altered CTCF binding activity on *PAX6* and *PAX6*-associated gene interactions

**Fig 4:**
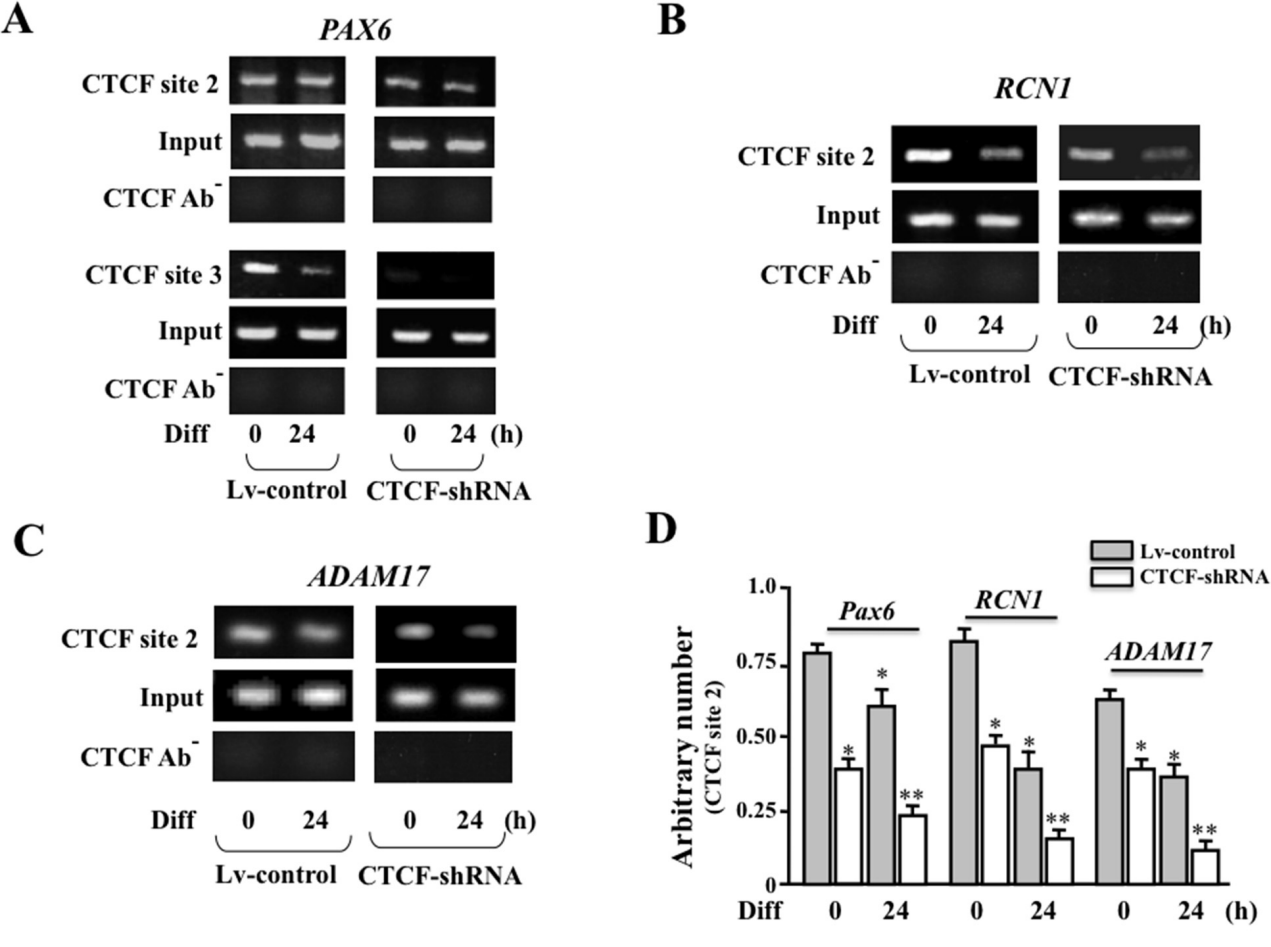
Effect of CTCF binding activity on interactions among *PAX6*, *RCN1* and *ADAM17* gene promoters. (***A***) Detection of CTCF binding activities at sites 2 and 3 in Pax6 promoter region by ChIP based PCRs in both lentivirus-infected Lv-control and CTCF-knocked down CTCF-shRNA cells during differentiation. (***B***) Detection of decreased CTCF-binding on site 2 of *RCN1* gene promoter during differentiation of both Lv-control and CTCF-shRNA cells. (***C***) Detection of decreased CTCF-binding on site 2 of *ADAM17* gene in differentiated Lv-control and CTCF -shRNA cells. (***D***) Statistic analysis of the significant decreases in CTCF binding in promoter regions of *PAX6*, *RCN1* and *ADAM17* genes in differentiated Lv-control and CTCF-shRNA cells, respectively. ChIP-based PCR was performed to amplify the selected CTCF bound DNA fragments in *PAX6*, *RCN1* and *ADAM17* promoter regions, respectively. Input and CTCF AB^-^ experiments were performed as controls with non-immunoprecipitated chromatins and in the absence of CTCF-specific antibody, respectively. Data were obtained from six independent ChIP and PCR experiments. Symbols “*” and “**” indicate significant differences between control and differentiated cells, control and CTCF-shRNA cells and differentiated control and CTCF-shRNA cells, respectively (*p*<0.05, n = 6).

To verify the specific effect of differentiation on CTCF binding capability in promoter regions of *PAX6*, *RCN1* and *ADAM17* in HTCE cells, we performed chromatin immunoprecipitation (ChIP) using specific antibodies against CTCF (Cell signaling). As it has been described in details of ChIP protocol in Methods, several pairs of the primers were used in ChIP-based PCRs for *PAX6*, *RCN1* and *ADAM17* to generate 250 to 300 bp DNA fragments, respectively. These primers as listed in Methods were designed according to predicted CTCF binding sites (using CTCFBSDB 2.0) upstream from *PAX6*, *RCN1* and *ADAM17* promoter regions. Results of ChIP-based PCR demonstrated that there were markedly weaker bands for *PAX6* (CTCF binding site 3), RCN1 and *ADAM17* in differentiation-induced HTCE lv-control cells that contain empty lentivirus only when the CTCF specific antibody was used to pull down the chromatins (left panels, *Fig 4A, 4B & 4C*). We found that there were a remarkable decreased CTCF binding in the *PAX6*, *RCN1* and *ADAM17* promoter regions in CTCF–shRNA HTCE cells. There were further diminished CTCF bindings in the *PAX6*, *RCN1* and *ADAM17* promoter regions in differentiation-induced CTCF-shRNA HTCE cells (right panels, *Fig 4A, 4B & 4C*). There is no significant alteration of CTCF binding on site 2 in comparison with site 3 of Pax6 (*Fig 4A*). For control experiments, non-immunoprecipitated chromatins were used as DNA templates in PCR experiments (labeled as input), and the additional control ChIP assays were performed by using rabbit antibody in immunoprecipitation (CTCF AB^-^) to elimination potential contaminations. ChIP and PCR results were finally quantized and plotted for statistical analysis shown in Fig 4D. The results from ChIP and PCR experiments indicate that there are CTCF-mediated interactions in *PAX6*, *RCN1* and *ADAM17* promoter regions very likely through CTCF binding to regulate these genes in chromatin remodeling in HTCE cell differentiation.

### Effect of CTCF on expressions of *PAX6* and *PAX6*-associated genes

**Fig 5:**
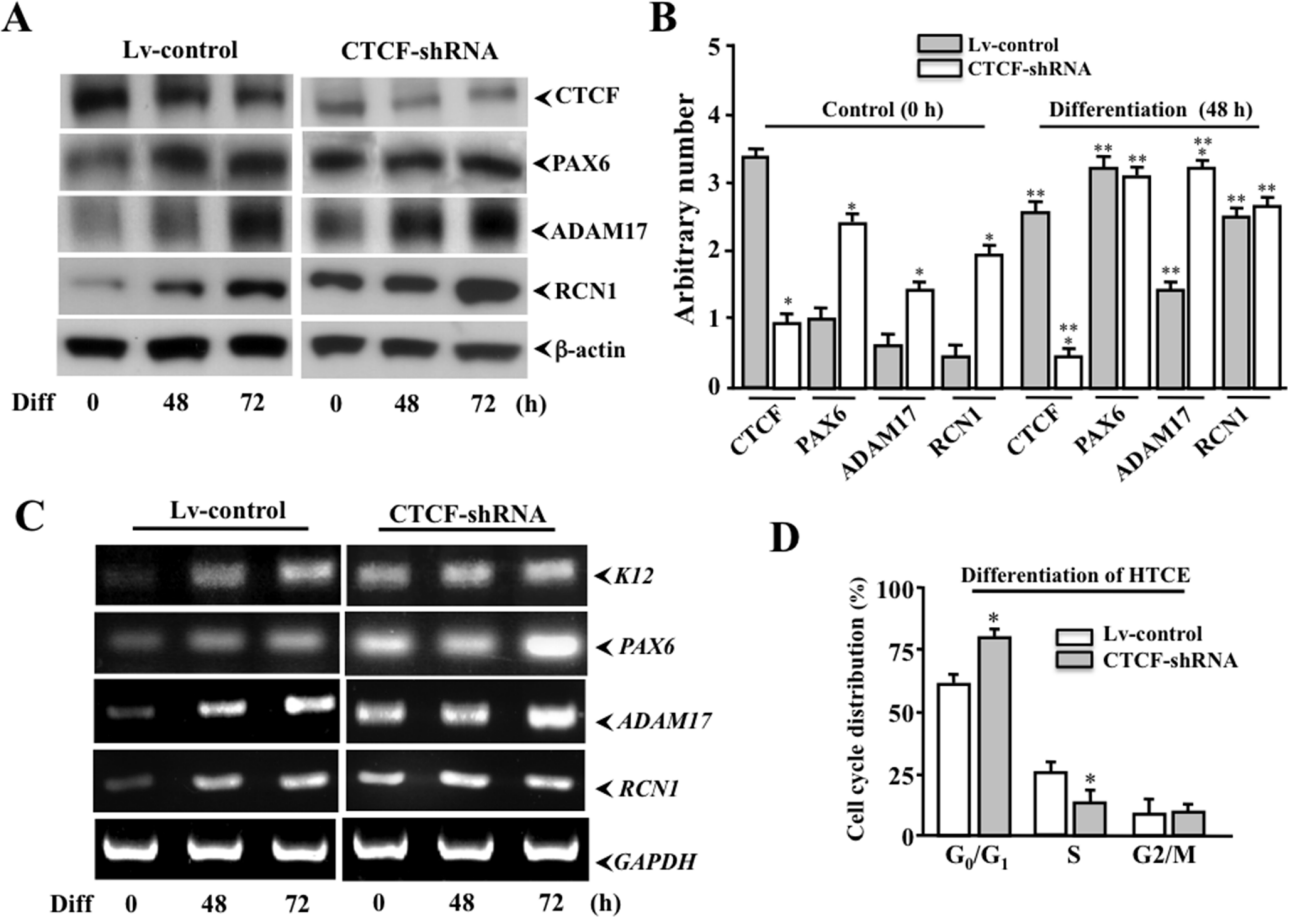
Effect of altered CTCF levels on expressions of *PAX6*, *RCN1* and *ADAM17*. (***A***) Expression patterns of CTCF, PAX6, ADAM17 and RCN1 proteins in Lv-control and CTCF mRNA knocked down CTCF-shRNA cells following a differentiation time course. (***B***) Statistical analysis of the effect of knocking down *CTCF* mRNA by *CTCF*-specific shRNA on expressions of CTCF, PAX6, ADAM17 and RCN1 proteins during HTCE cell differentiation at 48 h. (***C***) Comparison of expression in RNA levels of *K12*, *PAX6*, *ADAM17*, and *RCN1* in lentivirus-infected control and CTCF-shRNA cells following differentiation time courses. (***D***) Significant alteration of G_0_/G_1_ and S phases in cell cycle distribution of differentiation-induced CTCF-shRNA cells. Western blots and RT-qPCR were described in details in materials and methods. Treatments are as indicated. Cell cycle analysis was also described previously. Symbols “*” and “**” indicate the statistical significance between Lv-control and CTCF-shRNA cells before and after differentiation, respectively. Significant differences among groups were determined by One-way ANOVA and Tukey’s tests, and then Student’s *t* test was used to determine the significant difference between two samples at *P*<0.05 (n = 4 to 6).

In previous studies, we found that CTCF controls *PAX6* expression to regulate corneal epithelial cell proliferation/differentiation. In the present study, we first measured the correlation of CTCF, PAX6, ADAM17 and RCN1 expressions in differentiation of HTCE cells infected with Lv-control and CTCF-shRNA cells. There were decreases in CTCF expression and increases in PAX6, ADAM17 and RCN1 expression levels following a 72-hour differentiation time course in Lv-control cells, but not observed in CTCF mRNA-knocked down cells (*Fig 5A*). Quantitative analysis for the effect of differentiation on PAX6, ADAM17 and RCN1 protein expressions at 48 h were performed to determine significant changes by comparing Lv-control and CTCF-shRNA cells (*Fig 5B*). In CTCF-shRNA cells, CTCF expression was further diminished and PAX6, ADAM17 and RCN1 protein expressions were enhanced following the time course. Effects of altered CTCF activity on *PAX6* and *PAX6*-associated *ADAM17* and *RCN1* expression were further measured by RT-PCR in both Lv-control and CTCF-shRNA HTCE cells. There were increased transcription levels of K12, PAX6, ADAM17 and RCN1 mRNAs in HTCE cells with CTCF-shRNA following HTCE cell differentiation (*Fig 5C*). In addition, we also observed that population distribution of HTCE cells in the cell cycle was significantly altered by CTCF-shRNA showing an increase in G_0_/G_1_ phase and decrease in S phase (*Fig 5D*). The result is consistent with the data obtained from the previous study, suggesting that CTCF activation is required for corneal epithelial cell proliferation. Taken together, these results indicate that there is a functional role for CTCF to mediate interactions and expressions of *PAX6* and *PAX6*-associated genes in differentiation-induced HTCE cells.

## Discussion

In human corneal epithelial cells, growth factors elicit complex responses at early times by inducing specific cellular signaling pathways that transfer the signals to the nucleus and to activate transcription factor at later times [2,38,42,43,44]. In previous studies, we found that the epigenetic factor CTCF regulates corneal epithelial cell proliferation/differentiation in response to growth factor and stress stimulation to promote corneal epithelial wound healing [36,44]. CTCF plays a significant role in chromatin remodeling to coordinate groups of gene expression affecting cell fate. Altered CTCF activity results in abnormality of eye development including cornea, lens and retina by controlling eye-specific *PAX6* transcription by interaction with a repressor located in the promoter region of Pax6 gene [13,34,38]. However, it is unknown how the interaction of *PAX6* and differentiation-associated genes is regulated as a group by CTCF-mediated chromatin remodeling in corneal epithelial proliferation/differentiation. Studying molecular mechanism of corneal epithelial proliferation/differentiation can establish a significant foundation for understanding coordinated corneal epithelium-specific gene expression in corneal epithelial wound healing. Results presented in the study provided direct evidence for the first time to reveal CTCF-mediated chromatin alteration affecting *PAX6* and differentiation-associated gene transcription and expression as a group in corneal epithelial cell differentiation.

HTCE cells are telomerase-immortalized corneal epithelial cells and represent corneal epithelial progenitor characteristics [35]. HTCE cells maintain the intact cell cycle regulatory pathways, genomic stability and normal expression of differentiation-associated genes in the culture of defined keratinocyte-SFM medium containing a low calcium (90 μM) concentration. These cells are capable to differentiate and to generate a full thickness of multi-layered epithelium similar to the native corneal epithelium [35]. Our results showed that replacement of the low calcium and serum-free culture conditions effectively triggered HTCE cell differentiation evidenced by accumulation of cell population in the G_0_/G_1_ phases, reduced cells in S phase (*Fig 2A)* and increases in expressions of the corneal epithelium-specific keratins, including human corneal epithelium-specific K12 (*Fig 1*). During 96 h of differentiation induction period, we observed a correlated relationship in HTCE cells showing a decreased CTCF expression and increased PAX6 expression, which is consistent to the previous study results demonstrating CTCF suppresses *PAX6* expression by binding to a repressor located between the ectoderm enhancer and P_0_ promoter of *PAX6* gene [38].

Studies of differential gene expression profiling in HTCE cells require tremendous workload and largest numbers of database analysis. We spent our efforts focusing on the question of effects of CTCF-mediated chromatin alterations on interaction of eye-specific *PAX6* with differentiation-associated genes in these cells. The principal finding of this study is that the targeted genes involved in CTCF-mediated interactions in promoter region with *PAX6* during chromatin remodeling were identified by 4C technology and DNA microarray experiments. In current study, we focused on 2 differentiation-associated genes including *RCN1* and *ADAM17* found in 4C and DNA microarray approaches. The novel findings indicate that RCN1 and *ADAM17* genes interact with Pax6 promoter regions in a CTCF-mediated fashion through short and long ranges since *RCN1* and *ADAM17* are located in a homochromosomal and heterochromosomal position relative to the location of *PAX6* gene, respectively. CTCF-mediated interactions between *PAX6*, *RCN1* and *ADAM17* genes were further verified by FISH staining, suggesting that CTCF-mediated interactions of these genes very likely play functional roles in HTCE cell differentiation (*Fig 3).* Although FISH may sometimes underestimate frequency of the gene interactions, we believe that our results in the multiple accurate measurements with a high resolution have provided useful evidence for the actual interactions at chromatin levels of these genes. However, the other possibility that could not be excluded in this study is that transcription factors other than CTCF can also bind to the vicinity of CTCF binding site to indirectly mediate the interactions.

Results obtained in a pioneer 4C and DNA microarray experiments indicate that *PAX6* interacting with *RCN1* and *ADAM17* in the promoter regions is mediated by CTCF. CTCF mediates such interaction by binding to specific sites that were initially predicted by sequence analysis in the promoter regions of these genes (CTCF BSDB 2.1). To address this question, ChIP-PCR that is considered as a specific protein-DNA interaction detection approach was performed using CTCF-specific antibody to immunocoprecipitate and pull-down CTCF-bound *PAX6*, *RCN1* and *ADAM17* genes, and the specific effect of differentiation on the CTCF and these promoter interactions was examined. In addition, CTCF mRNA levels were knocked down by stable infection of lentiviral shRNA specific to CTCF in HCTE cells. These cells were also used in ChIP-PCR, cell cycle analysis and gene expression detection experiments. The comparison between CTCF-shRNA and control HTCE cells further elucidated the effect of CTCF on interactions of Pax6 and its interacting genes during HTCE cell differentiation.

## Conclusions

In conclusion, the present work demonstrates three important findings that are novel and very important for us to understand epigenetic regulation and molecular interaction in human corneal epithelial cell differentiation. *First*, there is a CTCF-mediated chromatin remodeling occurring in HTCE cell differentiation. CTCF regulates interactions of *PAX6* and differentiation associated genes through binding to specific DNA sites located in promoter regions of these genes. *Second*, the study results suggest that *PAX6* gene is one of the important eye-specific genes regulated by CTCF in chromatin remodeling, which is able to interact with differentiation-associated genes. In addition, the CTCF-mediated chromatin interactions between *PAX6* and differentiation-associated genes are in both homo-chromosomal and hetero-chromosomal levels. *Finally*, the functional significance of CTCF-mediated chromatin interactions between *PAX6* and differentiation-associated genes, such as *RCN1* and *ADAM17*, are verified during cell differentiation in CTCF-deficient HTCE cells determined by ChIP-PCR and cell cycle analysis. Further characterization of interaction between Pax6 and more differentiation-associated genes are required. Furthermore, more direct functional assays are also necessary to demonstrate effects of *PAX6* and these genes mediated by CTCF binding.
